# Environment and coordination of FeMo–co in the nitrogenase metallochaperone NafY†

**DOI:** 10.1039/d1cb00086a

**Published:** 2021-07-28

**Authors:** Aaron H. Phillips, Jose A. Hernandez, Lucía Payá-Tormo, Stefan Burén, Bruno Cuevas-Zuviría, Luis F. Pacios, Jeffrey G. Pelton, David E. Wemmer, Luis M. Rubio

**Affiliations:** St. Jude Children's Research Hospital Memphis TN 38105 USA; Department of Biochemistry and Molecular Genetics, College of Graduate Studies, Midwestern University Glendale AZ 85308 USA; Arizona College of Osteopathic Medicine, Midwestern University Glendale AZ 85308 USA; Centro de Biotecnología y Genómica de Plantas, Universidad Politécnica de Madrid, Instituto Nacional de Investigación y Tecnología Agraria y Alimentaria, Pozuelo de Alarcón Madrid 28223 Spain lm.rubio@upm.es; QB3 Institute, University of California Berkeley CA 94720 USA dewemmer@berkeley.edu; Division of Physical Biosciences of Lawrence Berkeley National Laboratory, University of California Berkeley CA 94720 USA; Department of Chemistry, University of California Berkeley CA 94720 USA

## Abstract

In nitrogenase biosynthesis, the iron-molybdenum cofactor (FeMo–co) is externally assembled at scaffold proteins and delivered to the NifDK nitrogenase component by the NafY metallochaperone. Here we have used nuclear magnetic resonance, molecular dynamics, and functional analysis to elucidate the environment and coordination of FeMo–co in NafY. H^121^ stands as the key FeMo–co ligand. Regions near FeMo–co diverge from H^121^ and include the η1, α1, α2 helical lobe and a narrow path between H^121^ and C^196^.

## Abstract 2

Broadening of NMR resonance spins used to map binding of paramagnetic FeMo–co to the nitrogenase metallocluster escort protein NafY.

## Graphical abstract

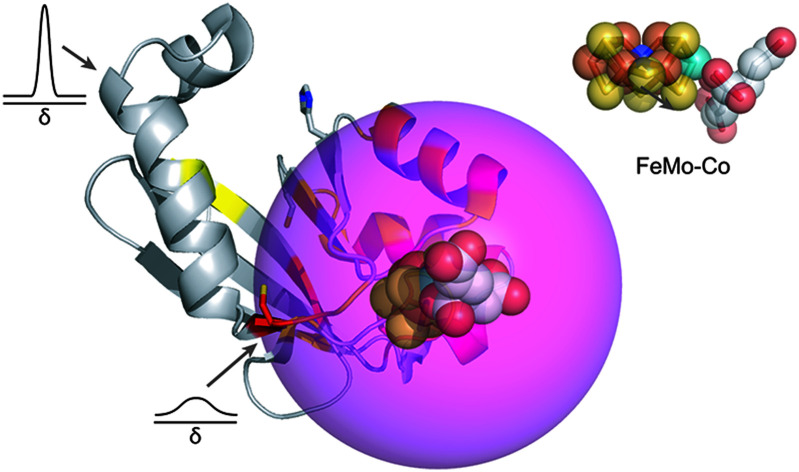

Metalloenzymes catalyze metabolic reactions in almost every physiological process and constitute one third of the proteome of a typical cell.^1,2^ Metallochaperones act as biochemical delivery services for metals or as escort proteins for labile metalloclusters from synthesis sites to target apo-enzymes, participating in their insertion into metalloenzymes in both prokaryotes^3^ and eukaryotes.^4^

Nitrogenase is the bacterial enzyme responsible for one-half of the world's nitrogen fixation.^5^ The majority of this biological nitrogen fixation is catalyzed by the molybdenum nitrogenase, composed of a dinitrogenase heterotetramer (NifDK), that reduces N_2_ to NH_3_, and a dinitrogenase reductase homodimer (NifH) that provides low potential electrons to NifDK.^6^ The iron-molybdenum cofactor (FeMo–co; [MoFe_7_S_9_C-homocitrate]) found at the active site of NifDK is assembled externally in a biosynthetic pathway including enzymes, molecular scaffolds and metallocluster escort proteins.^7^ At the end of this pathway, synthesized FeMo–co is specifically transferred from the NifEN scaffold protein to cofactor-less apo-NifDK by the NafY protein (Nitrogenase accessory factor Y).

Previous work showed that NafY consisted of two independently folding domains (Fig. S1, ESI†): an N-terminal domain (called N-NafY) responsible for binding apo-NifDK,^8^ and a C-terminal domain (called core-NafY) that binds FeMo–co with high affinity.^9,10^ These observations were consistent with the dual role of NafY as FeMo–co escort protein and as a molecular prop to stabilize apo-NifDK in a conformation for FeMo–co insertion.^11^ A structure of full-length NafY is not available, but N-NafY and core-NafY structures were solved by NMR^8^ and X-ray crystallography,^9^ respectively. N-NafY adopts an all α-helical fold while core-NafY folds as five-stranded β-sheet flanked by five α-helices. ^1^H–^15^N heteronuclear single quantum coherence (HSQC) spectra indicated N-NafY and core-NafY to have very weak interactions in intact NafY.^8^

The structural details of the interactions of NafY with FeMo–co and apo-NifDK are not well understood. For instance, there are no structural similarities of core-NafY to the FeMo–co binding pockets at NifEN or NifDK. In NifDK, FeMo–co is coordinated by one histidine and one cysteine residue.^12^ On the other hand, NafY site-directed mutagenesis of Cys and His residues only identified the surface exposed H^121^ as involved in FeMo–co binding.^10^ H^121^ is part of a His-Phe-Gly motif conserved in NafY homologs, such as NifB and NifX, that bind a structurally similar FeMo–co biosynthetic precursor called NifB-co.^11^

It is worth noting that characterization of nitrogenase maturation is crucial for a thorough understanding of nitrogenase activity in cells, and that NafY characterization stands of particular interest as a tool to study FeMo–co synthesis and NifDK maturation in the biotechnology context of generating plants capable of fixing their own nitrogen.^13^

This study sheds light on the coordination and environment of FeMo–co in NafY. Using NMR spectroscopy analysis, we assigned 108 amide resonances out of the possible 125 non-proline residues present in core-NafY (from R^100^ to F^231^ of NafY) and then collected ^1^H–^15^N HSQC spectra of core-NafY in complex with FeMo–co. Core-NafY was prepared (Fig. S2, ESI†) and loaded with FeMo–co as previously described,^9^ with the modification that the *Escherichia coli* cells were grown in isotopically labeled media according to Marley *et al.*^14^ Resonance assignments for most residues of core-NafY were obtained with 3D ^15^N-resolved nuclear Overhauser effect spectroscopy (NOESY), HNCA, CBCAcoNH and HNCACB spectra. The level of attenuation of each resonance was determined by comparing the intensity of ^1^H–^15^N HSQC spectra in the presence and absence of FeMo–co.

The resonances of spins that are in close proximity to the paramagnetic FeMo–co were attenuated due to the enhanced relaxation caused by the presence of unpaired electron spins^15^ thereby allowing localization of the FeMo–co binding site. The overlaid spectra and a depiction of residues affected by FeMo–co paramagnetism are shown in Fig. 1 and Table S1, ESI.†

**Fig. 1:**
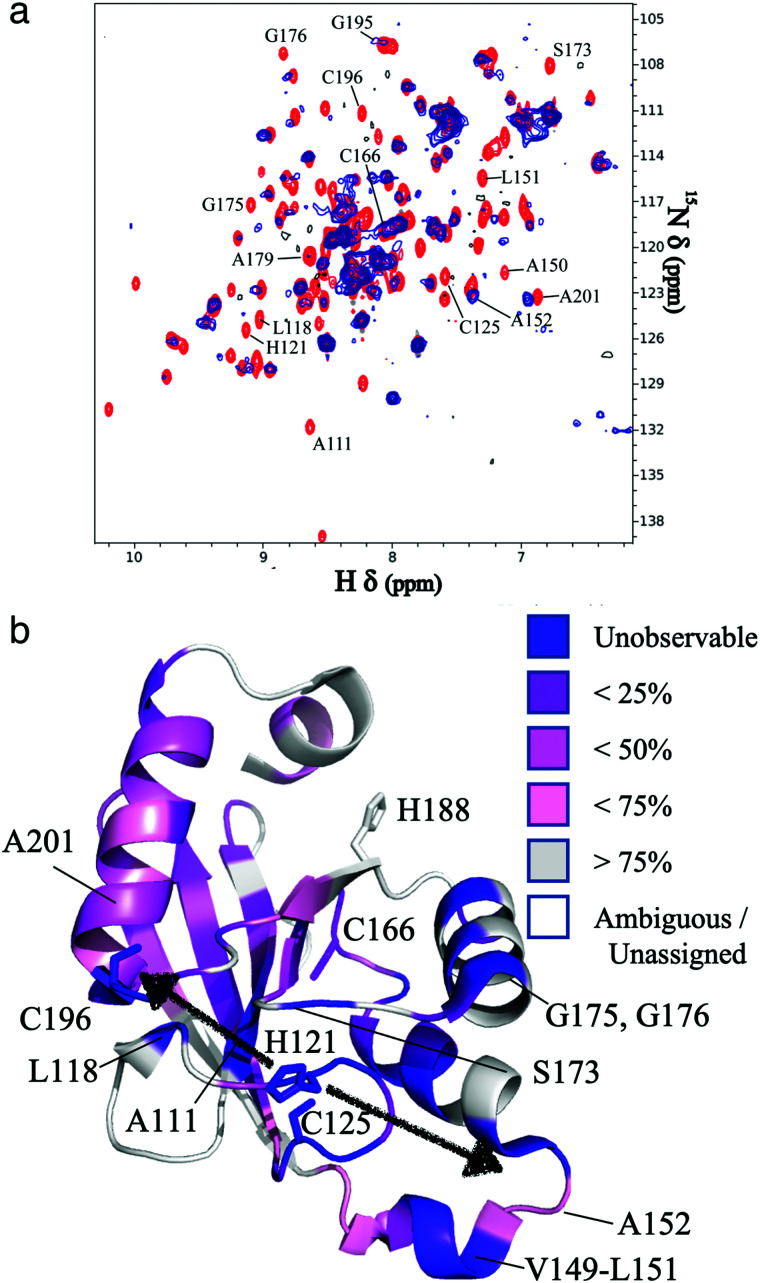
Localization of the FeMo–co binding site in core-NafY. (a) Overlaid ^1^H–^15^N HSQC spectra of apo-core-NafY (red) and core-NafY complexed with FeMo–co (blue). (b) Crystal structure of core-NafY (PDB 1P90) color coded by the level of attenuation upon FeMo–co binding. The increments of color coding are half standard deviations about the mean denoted by setting the mean to 50%. Black arrows around H^121^ indicate possible FeMo–co locations. Several relevant residues are labeled.

Three potential FeMo–co binding residues (H^121^, C^125^ and C^196^) displayed remarkable broadening of NMR resonances, indicating proximity to FeMo–co. We note that attenuation did not necessarily imply interaction. Strong attenuation of H^121^ amide resonance is consistent with previously demonstrated importance in FeMo–co coordination.^10^ Regions affected by FeMo–co are mostly narrowed down to an α helical lobe formed by residues 149 to 162 and a narrow path between H^121^ and C^196^ involving A^111^, L^118^, S^173^, and G^195^. Broadening of C^125^ resonance indicated location in the vicinity of FeMo–co. However, the C125A variant has been shown to retain FeMo–co binding,^10^ and the structure shows that its thiol group is not properly oriented towards H^121^. The C^196^ thiol group is located 12 Å away from H^121^ but it is not properly oriented in the crystal structure to allow FeMo–co coordination by these two residues. Neither C^125^ nor C^196^ are conserved among NafY homologues. Putative metal binding residues H^188^ and C^166^ do not show strong attenuation consistent with previously reported unimportance in FeMo–co binding.^10^ The above mentioned α helical lobe contains the strongly attenuated D^154^ residue, with its β-carboxyl group oriented to the surface and could potentially interact with FeMo–co. However, the distance between H^121^ and D^154^ is 22 Å precluding concerted binding.

Molecular modelling based upon protein–ligand docking searches for possible FeMo–co binding sites in core-NafY crystal structure (1P90) suggested a putative site in an exposed cleft defined by residues 117–119, 121, 172, and 193–196 (Fig. S3, ESI†). H^121^ and C^196^ are at opposite ends of that site although their side chains are not properly oriented towards the inner part of the cleft. Interestingly, energy minimizations of the structure in complex with FeMo–co in that site led to H^121^ and C^196^ side chain rotations towards the Mo and Fe1 terminal atoms of FeMo–co, respectively (Fig. S3, ESI†). Neither electrostatic potential analysis nor surface topography supports FeMo–co docking at the surface between H^121^–C^125^ or H^121^–C^166^ (Fig. S4, ESI†). The possible contribution of N-NafY was not considered in these models as previous NMR study showed that NafY domains tumbled independently.^8^ Structural models of full length NafY generated with Robetta concur with the prediction of an architecture with separated domains (Fig. S5, ESI†).

The optimized structure of core-NafY with FeMo–co between H^121^ and C^196^ was used as initial geometry for all-atom molecular dynamics (MD) exploratory 10 ns simulations aimed at probing complex stability. Various secondary structure elements changed along the simulation. Two tiny β-strands 117 + 118 and 196 + 197, as well as the 3_10_ helix 147–151 identified in the crystal structure, vanished and instead two initial loop segments 122–124 and 217–219 adopted 3_10_ helix conformations (Fig. S6, ESI†). These conformational variations are usually regarded as transient substructures associated with protein backbone dynamics. MD calculations yielded the final structure shown in Fig. 2a, where FeMo–co is predicted to locate stably in the cleft, with the Nδ1 atom of H^121^ and the Sγ atom of C^196^ properly oriented towards the Mo and Fe1 atoms at distances around 2.9 and 2.2 Å, respectively (inset box of Fig. 2a). The Oγ atom of S^124^ forms a hydrogen bond with the C6 carboxyl group of homocitrate (Fig. S7, ESI†). The longer than expected H^121^·Nδ1–Mo distance could be due to suboptimal FeMo–co parameters. The distance between Nδ1 atom of H^121^ and Sγ atom of C^196^ varied between 11.6 and 12.0 Å along the simulation (Fig. 2b), with a final value of 11.86 Å. In contrast, the Sγ atom of C^125^ was at an average distance of 11.95 Å to the closest Fe6 atom of FeMo–co (Fig. S8, ESI†) and was not properly oriented to coordinate it. The NafY protein backbone fluctuated little with root mean square fluctuation (RMSF) values below 1.5 Å in all residues except V^149^ (2.14 Å), K^194^ (2.03 Å) and the flexible N- and C-ends (Fig. 2c). It must be stressed that residues in the vicinity of H^121^ exhibited particularly low fluctuation with values even lower than 1.0 Å despite being in loops.

**Fig. 2:**
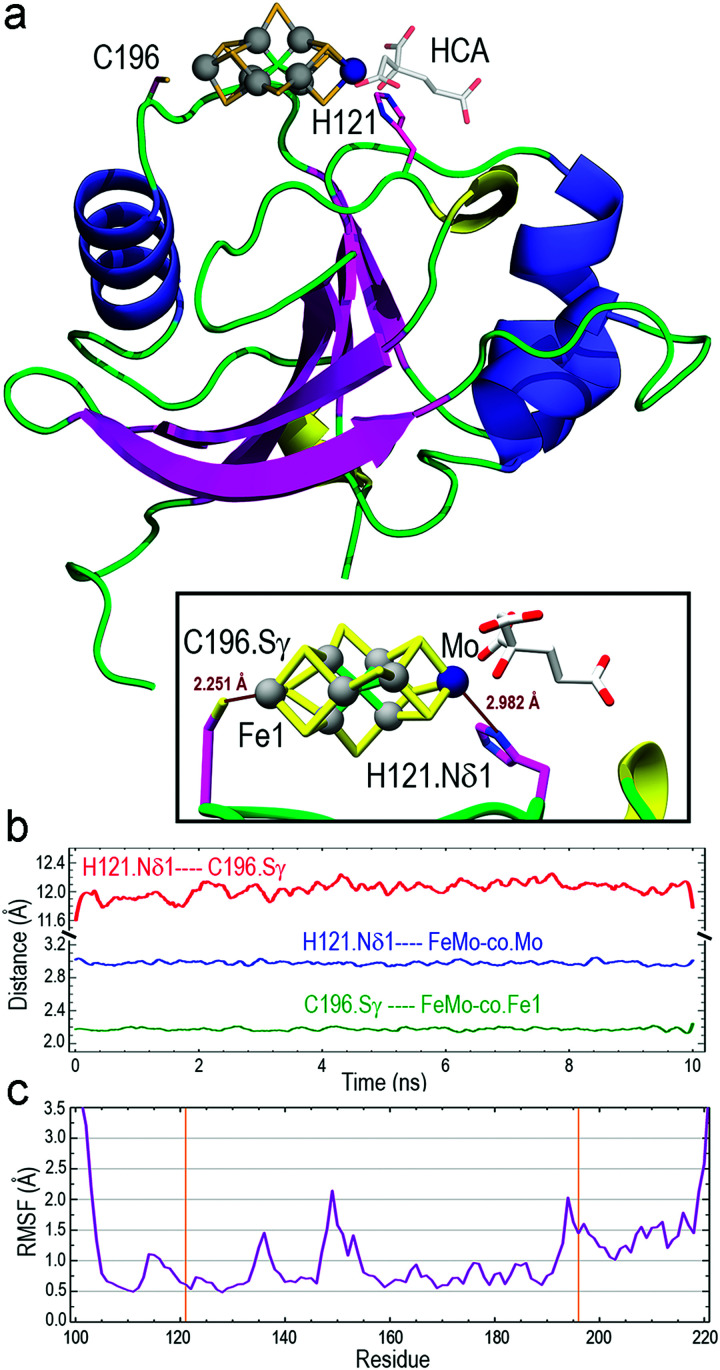
Results of all-atom MD 10 ns simulations of the core-NafY FeMo–co complex. (a) Final complex structure with ribbons colored by secondary structure: α-helices in slate blue, 3_10_ helices in yellow, and β-strands in magenta. The inset box shows the binding site with H^121^·Nδ1–FeMo–co·Mo and C^196^·Sγ-FeMo–co–Fe1 final distances. (b) Change along the MD simulation of H^121^·Nδ1–C^196^·Sγ, H^121^·Nδ1–FeMo–co·Mo, and C^196^·Sγ–FeMo–co–Fe1 distances. (c) Root mean square fluctuation of Cα atoms. Orange vertical lines indicate H^121^ and C^196^ residues.

The contribution of C^196^ to NafY function was investigated by generating a C196A full-length NafY variant and assessing *in vitro* binding to FeMo–co and apo-NifDK. In addition, H121L and H121L/C196A NafY variants were generated, purified, and assayed in parallel (Tables S2–S5 and Fig. S9, ESI†). FeMo–co binding to NafY is known to alter its migration in anoxic native gels. The effect of FeMo–co on migration of full-length NafY wild-type and variants is shown in Fig. 3. In contrast to H121L, the C196A variant showed wild-type migration pattern suggesting it had no critical role in FeMo–co binding or, at least, that its contribution was weaker than the affinity threshold detected in gel migration experiments. On the other hand, the H121L/C196A double mutant gel migration was the same as the H121L variant. In addition, apo-NifDK binding was tested in pull-down experiments in which Strep-tagged NafY variants served as bait to recover apo-NifDK from *A. vinelandii* UW146 (Δ*nifB*Δ*nafY*) protein extracts. Comparable amounts of apo-NifDK were recovered with NafY wild type and variants (Fig. S10, ESI†), consistent with the fact that apo-NifDK binding is mainly mediated by the N-NafY domain.^8^

**Fig. 3:**
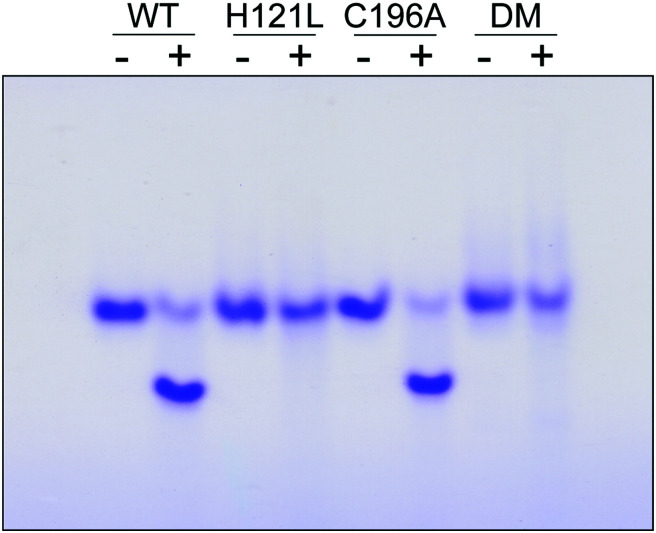
Analysis of NafY variant interaction with FeMo–co. Anoxic native gel stained with Coomassie, showing the shift in NafY mobility upon FeMo–co binding. Symbols above lanes indicate NafY samples without (−) or with (+) FeMo–co. DM, H121L/C196A double mutant.

To understand why H^121^ but not C^196^ was important for NafY/FeMo–co complex stability, the initial exploratory MD calculations (“trajectory 1”) were supplemented with two additional simulations restraining in input only the presence of H^121^ (“trajectory 2”) or only the presence of C^196^ (“trajectory 3”). The results revealed that the presence of H^121^ suffices to keep FeMo–co bound in the site whereas releasing this restraint in exchange for restraining only the presence of C^196^ gave place to unstable FeMo–co geometry, which moved nearly freely while “anchored” at C^196^. In fact, trajectory 1 and trajectory 2 results were nearly indistinguishable regarding the H^121^·Nδ1–C^196^·Sγ distance while trajectory 3 showed very large variations. Since RMSF values of the protein backbone were similar in the three trajectories, those large distance variations between H^121^ and C^196^ correspond to large motions of side chains (Table S6 and Fig. S11, ESI†). Finally, root mean square deviation (RMSD) results for trajectory 2 showed that the single restraint of the presence of H^121^ yields the lowest mobilities of core-NafY and FeMo–co (Table S6, ESI†).

Thus, MD simulations suggest that core-NafY binds FeMo–co in similar fashion to NifDK, *i.e.* through His and Cys residues. The critical role of H^121^ for complex stability compared to C^196^ (Fig. 3) suggests it provides the initial and highest affinity coordination to FeMo–co. Surprisingly, the H121L and the H121L/C196A variants retain some capacity to activate apo-NifDK with FeMo–co (Fig. S12, ESI†), even though their complexes are not sufficiently strong to survive anoxic gel electrophoresis. Since NafY/FeMo–co complex preparation involved multiple cycles of concentration/dilution (Material and methods, ESI†) it is unlikely that this activation was due to unbound FeMo–co in the samples. It could rather result from FeMo–co being weakly attached to NafY independently of H^121^, for instance at the α helical lobe detected by NMR signal attenuation. The physiological relevance of this observation is not known. Because NafY receives FeMo–co from the NifEN scaffold and delivers it to apo-NifDK a dual FeMo–co binding mode would be possible. Clearly, the different techniques used here have different thresholds to observe the NafY/FeMo–co interaction.

NMR spectroscopy has often been used to analyze interactions between metal ions and proteins^16,17^ and to characterize the transfer of iron–sulfur clusters from scaffold proteins to target apo-proteins.^18,19^ Here we have used NMR spectroscopy to further define the region of NafY that interacts with FeMo–co. NMR spectroscopy shows proximity of the cofactor to two regions in NafY: a helical lobe consisting of η1, α1, α2 and the loop between β2 and β3, and a narrow path between H^121^ and C^196^. In agreement with NMR data, *in silico* docking simulations indicates that FeMo–co coordination by H^121^ and C^196^ is possible. However, there is no direct biochemical evidence to support this coordination mode as C^196^ is neither required for apo-NifDK activation nor essential to the stability of the NafY/FeMo–co complex. To explain this paradox, we propose a mechanism in which H^121^ provides the key binding site and C^196^ would provide a second, stabilizing ligand.

Funds for the 900 MHz NMR spectrometer were provided by the NIH through grant GM68933 to D. E. W. This work was also funded by FEDER/Ministerio de Ciencia, Innovación y Universidades-Agencia Estatal de Investigación grant 2017-88475-R (L. M. R), by Severo Ochoa Program for Centres of Excellence in R&D from Agencia Estatal de Investigación of Spain grant SEV-2016-0672 to the CBGP, and by Midwestern University intramural funds (J. A. H.). L. P.-T. is recipient of FPU16/02284 from Ministerio de Ciencia, Innovación y Universidades. We thank Paul W. Ludden for support, and Kaya W. Erbil for discussions.

## Conflicts of interest

There are no conflicts to declare.

## Supplementary Material

CB-002-D1CB00086A-s001

## References

[cit1] Dupont C. L., Yang S., Palenik B., Bourne P. E. (2006). Proc. Natl. Acad. Sci. U. S. A..

[cit2] Waldron K. J., Rutherford J. C., Ford D., Robinson N. J. (2009). Nature.

[cit3] Py B., Barras F. (2010). Nat. Rev. Microbiol..

[cit4] Philpott C. C., Ryu M. S., Frey A., Patel S. (2017). J. Biol. Chem..

[cit5] Fowler D., Coyle M., Skiba U., Sutton M. A., Cape J. N., Reis S., Sheppard L. J., Jenkins A., Grizzetti B., Galloway J. N., Vitousek P., Leach A., Bouwman A. F., Butterbach-Bahl K., Dentener F., Stevenson D., Amann M., Voss M. (2013). Philos. Trans. R. Soc., B.

[cit6] Seefeldt L. C., Yang Z. Y., Lukoyanov D. A., Harris D. F., Dean D. R., Raugei S., Hoffman B. M. (2020). Chem. Rev..

[cit7] Buren S., Jimenez-Vicente E., Echavarri-Erasun C., Rubio L. M. (2020). Chem. Rev..

[cit8] Hernandez J. A., Phillips A. H., Erbil W. K., Zhao D., Demuez M., Zeymer C., Pelton J. G., Wemmer D. E., Rubio L. M. (2011). J. Biol. Chem..

[cit9] Dyer D. H., Rubio L. M., Thoden J. B., Holden H. M., Ludden P. W., Rayment I. (2003). J. Biol. Chem..

[cit10] Rubio L. M., Singer S. W., Ludden P. W. (2004). J. Biol. Chem..

[cit11] Rubio L. M., Rangaraj P., Homer M. J., Roberts G. P., Ludden P. W. (2002). J. Biol. Chem..

[cit12] Einsle O., Tezcan F. A., Andrade S. L., Schmid B., Yoshida M., Howard J. B., Rees D. C. (2002). Science.

[cit13] Buren S., Young E. M., Sweeny E. A., Lopez-Torrejon G., Veldhuizen M., Voigt C. A., Rubio L. M. (2017). ACS Synth. Biol..

[cit14] Marley J., Lu M., Bracken C. (2001). J. Biomol. NMR.

[cit15] Bertini I., Luchinat C., Piccioli M. (2001). Methods Enzymol..

[cit16] Piccioli M. (2020). Magnetochemistry.

[cit17] Wishart D. S., Sykes B. D. (1994). J. Biomol. NMR.

[cit18] Banci L., Brancaccio D., Ciofi-Baffoni S., Del Conte R., Gadepalli R., Mikolajczyk M., Neri S., Piccioli M., Winkelmann J. (2014). Proc. Natl. Acad. Sci. U. S. A..

[cit19] Jensen M. R., Hass M. A., Hansen D. F., Led J. J. (2007). Cell. Mol. Life Sci..

