# Supplementary material for: Environment and coordination of FeMo–co in the nitrogenase metallochaperone NafY
Source: RSC Chem Biol. 2021 Jul 28;2(5):1462–5. doi: 10.1039/d1cb00086a (PMC8496260; doi:10.1039/d1cb00086a)
Supplement: CB-002-D1CB00086A-s001 [file CB-002-D1CB00086A-s001.pdf]

Electronic Supplementary Material (ESI) for RSC Chemical Biology.
This journal is © The Royal Society of Chemistry 2021

SUPPORTING INFORMATION

Environment and coordination of FeMo-co in the nitrogenase metallochaperone NafY

Aaron Phillips,[†a] Jose A. Hernandez,[†bc] Lucía Payá-Tormo,[d] Stefan Burén,[d] Bruno Cuevas-Zuviría,[d] Luis F. Pacios,[d] Jeffrey G. Pelton,[ef] David E. Wemmer[*efg] and Luis M. Rubio[*d]

[a] St. Jude Children's Research Hospital, Memphis, TN 38105, USA
[b] Department of Biochemistry and Molecular Genetics, College of Graduate Studies, Midwestern University, Glendale, AZ 85308, USA
[c] Arizona College of Osteopathic Medicine, Midwestern University, Glendale, AZ 85308, USA
[d] Centro de Biotecnología y Genómica de Plantas, Universidad Politécnica de Madrid, Instituto Nacional de Investigación y Tecnología Agraria y Alimentaria, Pozuelo de Alarcón, 28223 Madrid, Spain
E-mail: lm.rubio@upm.es
[e] QB3 Institute, University of California, Berkeley, CA 94720, USA
[f] Division of Physical Biosciences of Lawrence Berkeley National Laboratory, University of California, Berkeley, CA 94720, USA
[g] Department of Chemistry, University of California, Berkeley, CA 94720, USA
E-mail: dewemmer@berkeley.edu
† These authors contributed equally. *Corresponding authors.

TABLE OF CONTENTS

Materials and methods S2
Table S1: Attenuation of spins resonances in core-NafY upon FeMo-co binding S6
Table S2: NafY wild type (WT) and site-directed variants generated for this work S7
Table S3: Primer sequences used for overlapping PCR reactions S7
Table S4: Primer combinations and PCR conditions for PCR 1 and PCR 2 S7
Table S5: Purified NafY variants used in this study S7
Table S6: Mean root mean square deviations values (RMSD) and the corresponding standard deviations (σ) along MD simulations for the three MD trajectories of the core-NafY FeMo-co complex S8
Fig. S1: Schematic representation of NafY domains S9
Fig. S2: SDS-PAGE analysis of ^{15}N-core-NafY purification steps S9
Fig. S3: Binding site in core-NafY located in molecular modelling S10
Fig. S4: Poisson-Boltzmann electrostatic potentials S11
Fig. S5: Model structure of the complete 243-amino acids NafY protein S12
Fig. S6: Secondary structure of core-NafY identified with DSSP S13
Fig. S7: Neighborhood of 4 Å around any atom of homocitrate S13
Fig. S8: Change of C^{125}.Sγ – FeMo-co.Fe6 distance S14
Fig. S9: SDS-PAGE analysis of NafY variants purification processes S14
Fig. S10: Analysis of NafY variant interaction with apo-NifDK S15
Fig. S11: Comparison of output for the three MD trajectories of the core-NafY/FeMo-co complex starting at the following initial structures S15
Fig. S12: Activation of apo-NifDK by NafY/FeMo-co complexes S16
References S17

MATERIALS AND METHODS

All chemicals used were of analytical grade and were used as received from the chemical manufacturer, unless otherwise indicated. Ultrapure water used in all experiments was from a Millipore system.

Bacterial strains and growth conditions

Escherichia coli DH5α and BL21 (pREP-4) strains were grown in LB medium with shaking (220-250 rpm) at 37°C. For growth on solid medium, 1.5% agar was added to the growth culture. Antibiotics were used at standard concentrations[1]. Overexpression of GST-core-NafY (Fig. S2) was induced by addition of 1 mM IPTG to the culture medium and incubation for 3 h. Isotopic labeling was performed according to Marley[2].

E. coli BL21 (DE3) strains 2161, 2162, 2163 and 2164 were used as host to express NafY wild-type, H121L, C196A and H121L/C196A, respectively. Cultures of 100 ml were grown in LB media supplemented with ampicillin (in 500 ml flasks) at 37°C and 200 rpm until the cultures reached an optical density (OD_{600}) of 0.8-1.0. Cultures were used to inoculate 1 L LB medium supplemented with ampicillin (in 4 L flasks) at an initial OD_{600} of 0.02-0.05. Cells were grown at 37°C and 200 rpm until the culture reached an OD_{600} of 0.6-0.8, then induced by addition of 3 g/L lactose and cultured overnight at 30°C and 105 rpm. The cells were collected by centrifugation at 4,500 rpm for 10 min at 4°C, frozen in liquid N_2 and stored at -80°C.

Site-directed mutagenesis and cloning of Twin-Strep-tagged NafY variants

NafY variants (Table S2) were expressed from a modified pET-16b where the 10xHis-Factor Xa site had been replaced by a Twin-Strep-TagII-TEV sequence (pN2LP29). Introduction of site-directed mutations in *Azotobacter vinelandii nafY* was carried out by overlapping PCR using primers listed in Table S3 as described [3]. Primer combinations and PCR conditions are listed in Table S4. Plasmid pRHB62 (with the *A. vinelandii nafY* wild-type gene inserted into a pGEX-4T-3 plasmid for expression of GST-tagged NafY [4]) was used as the template to generate DNA fragments for the overlapping PCR reactions (PCR 1 and 2). The overlapping PCR was performed using primers 2660 (5'-TCTTTATTTTCAAGGTCATATGGTAACCCCCGTGAACATG-3´) and 2661 (5´-CGGGCTTTGTTAGCAGCCGGATCCTCATGCCCTGGCCGCC-3´) as external primers (Tm 65.5°C), generating *nafY* gene variants with 19 bp and 24 bp extensions (5' and 3' respectively) complementary to the cloning vector. PCR reactions were carried out using Phusion™ High-Fidelity DNA Polymerase (Thermo Scientific) according to manufacturer recommendations. The *nafY* gene fragments were inserted into plasmid pN2LP29 digested with *NdeI* and *BamHI* by Exonuclease and Ligation-Independent Cloning (ELIC) [5]. DNA of digested vector and *nafY* gene variants were mixed in a 1:4 ratio and incubated at room temperature for 10 min, and then transformed into *E. coli* DH5α. All *nafY* variants were confirmed by DNA sequencing.

Purification of ^{15}N labeled core-NafY

The ^{15}N-GST-tagged core-NafY protein was purified in aerobic conditions from cell-free extracts of induced *E. coli* BL21 (pREP-4) cells transformed with plasmid pRHB263 (P_{tac}-GST-core-NafY) [6]. Cell pellets were resuspended in buffer A (10 mM sodium phosphate, 1.8 mM potassium phosphate (pH 7.3), 140 mM NaCl, and 2.7 mM KCl) and disrupted at 12,000 psi using a French press. Cell lysates were clarified by two consecutive centrifugation steps at 25,000 x *g* for 40 min each. The cell-free extract was loaded onto a 25-ml GSH-Sepharose Fast Flow affinity column (GE Healthcare) and washed with 250 ml of

buffer A supplemented with 1% Triton X-100. The GST-tagged protein was eluted from the column by applying 75 ml of buffer B (50 mM Tris-HCl (pH 8.0) and 10 mM GSH). The GST-tag was cleaved by the addition of 10 μg of purified TEV protease/mg of GST-tagged protein, followed by 2 h incubation at 30°C. The protease reaction mixture was then subjected to three sequential chromatographic steps: *(i)* Ni^{2+} affinity (to remove TEV protease), *(ii)* gel filtration on Sephadex G-25 (to remove GSH), and *(iii)* GSH-Sepharose (to remove the free GST tag). A typical purification protocol yielded 0.2 mg of ^{15}N-core-NafY per gram of *E. coli* cell paste. Protein purity was estimated to be >95% based on SDS-PAGE analysis (Fig. S2). Purified ^{15}N-core-NafY was concentrated by ultrafiltration through a YM10 membrane (Millipore) using an Amicon cell device to 0.6 mM for nuclear magnetic resonance analysis.

Anaerobic purifications of NafY variants for FeMo-co binding and apo-NifDK activation assays

Purification procedures aimed to assay NafY activities were carried out under anaerobic conditions. Buffers were previously made anaerobic by sparging with N_2. Each NafY variant was isolated from about 25 g of *E. coli* cells. Cells were resuspended in 50 ml buffer A (100 mM Tris-HCl (pH 8.2), 200 mM NaCl, 10% glycerol, 2 mM dithionite (DTH)) supplemented with 1 mM phenylmethylsulfonyl fluoride, 1 μg/ml leupeptin and 5 μg/ml DNase I. Cells were lysed in an Emulsiflex-C5 homogenizer (Avestin Inc.) at 15,000 psi. Cell-free extracts (CFE) were prepared by centrifugation of the lysates for 1 h at 50,000 x *g* (4°C).

Purifications were performed inside a glovebox (Coy Laboratory) (<5 ppm of O_2) using Strep-binding affinity chromatography. The CFE was loaded using a peristaltic pump (Masterflex C/L, Cole Parmer) at 2 ml/min into a 5-ml Strep-Tactin XT Superflow Cartridge (IBA Life Science) previously equilibrated with buffer A. The NafY proteins were eluted, following 5 consecutive washes of 15 ml buffer A, with 15 ml of buffer A supplemented with 50 mM biotin, concentrated using 10 kDa cut-off centrifugal membrane filters (Amicon Ultra-15, Millipore), and desalted using a PD-10 desalting column (GE Healthcare). NafY proteins were analyzed by SDS- gels stained with Coomassie blue and stored as protein beads in liquid N_2.

Apo-NifDK activation assays

NafY/FeMo-co complexes were generated by incubating 10 μg of NafY and FeMo-co (2-fold molar excess) in 150 μl of anaerobic 22 mM Tris-HCl buffer (pH 7.4), 2 mM DTH for 5 min on ice followed by 20 min at room temperature. Samples were supplemented with anaerobic buffer to 500 μl total volume and concentration down to 100 μl using a 10-kDa cut-off centrifugal membrane filters. Samples were subjected to four additional cycles of concentration/dilution and were left at 100 μl final volume to decrease unbound FeMo-co 2,000-fold.

Apo-NifDK activation samples were prepared in rubber-stopped 9-ml anaerobic vials and included: 0.6 μM apo-NifDK, 1.2 μM NafY or NafY/FeMo-co complex, 0.1 mg BSA, and ATP-regenerating mixture (1.23 mM ATP, 18 mM phosphocreatine, 2.2 mM $MgCl_2$,3 mM DTH and 46 μg/ml of creatine phosphokinase, 22 mM Tris-HCl pH 7.5) in a final volume of 600 μl. Reactions were incubated for 30 min at room temperature after which 24 μM NifH was added, N_2 was replaced by Ar/acetylene (95%/5%) and nitrogenase activity proceeded for 30 min at 30°C.

Preparation of *A. vinelandii* UW146 cell-free extracts and study of interaction between apo-NifDK and NafY variants

Extracts of *A. vinelandii* UW146 (*ΔnifB ΔnafY*) were used to study the interaction between NafY variants and apo-NifDK (Fig. S10). Twenty-eight g of *A. vinelandii* UW146 cells were resuspended in 50 ml of the above-described buffer A. Soluble cell-free extracts were prepared as described above.

StrepTactin-XT resin (IBA Life Science) saturated with Twin-Strep tagged NafY variants (or without NafY as control) were incubated with 10 ml of UW146 CFE for 3 h (room temperature) with mild agitation. Following transfer to empty columns for gravity chromatography, resin was washed with 35 ml buffer A. Proteins were eluted using 3.5 ml of buffer A supplemented with 50 mM biotin. Eluted proteins were concentrated to 1.5 ml using 10 kDa cut-off spin filters (Microcon, Millipore). Protein interactions were analyzed by Coomassie blue staining of SDS-PAGE gels, and stored under anaerobic conditions at -80°C.

Anoxic Native Gel Electrophoresis of NafY/FeMo-co complexes

To generate complexes, 10 µg of each purified Twin-Strep-NafY variant was incubated with 0.625 nmol FeMo-co (2-fold excess) in 150 µl of 22 mM Tris-HCl buffer (pH 7.4), 15% glycerol, 2 mM DTH for 5 min on ice followed by 20 min at room temperature. Proteins (50 µl samples) were separated by anoxic native gel electrophoresis (7–16% acrylamide, 0–20% sucrose gradient gels) under anaerobic conditions in a glovebox (Coy Laboratory) as described[7, 8]. Gels were casted under aerobic conditions and previously made anaerobic by electrophoresis for 2 h at 100 V (4°C). Electrophoresis run at 4°C under constant voltage for 1800 Vh. Gels were stained with Coomassie.

NMR Spectroscopy

NMR samples of ^{15}N-core-NafY were obtained by concentrating purified protein in buffer A supplemented with 0.02% (w/v) NaN_3 in either 100% ^2H_2O or 10% (v/v) ^2H_2O to a 0.6 mM protein concentration. ^{15}N-core-NafY NMR data sets were collected on a 135-amino acid polypeptide containing 132 residues of NafY (Arg^{100}-Phe^{231}) plus three N-terminal residues (Gly-His-Met) introduced during the cloning procedure (Figure S1). NMR experiments were recorded at 298 K on a Bruker Avance II 900 equipped with a CPTXI cryoprobe. NMR data were processed using NMRPipe or rNMRtk, and spectra were analyzed with NMRDraw [9] and CARA [10].

^{15}N-core-NafY/FeMo-co complex preparation for NMR determinations

FeMo-co was purified as previously described [11]. All glassware was rinsed with 4 N HCl overnight to remove traces of contaminant metals, and then rinsed thoroughly with deionized water. All steps of the preparation were performed inside an anaerobic (<5 ppm O_2) glove box (Coy) with a 95% N_2/5% H_2 environment. ^{15}N-core-NafY was pre-treated with 1.5 mM DTH in anaerobic buffer C (10 mM sodium phosphate, 1.8 mM potassium phosphate (pH 7.3), 140 mM NaCl, and 2.7 mM KCl) for 30 min. FeMo-co was added to purified ^{15}N-core-NafY in a 1.5 mol of purified FeMo-co to 1 mol of protein ratio in 1 ml of anaerobic buffer A. The mixture was incubated at room temperature for 15 min. The unbound FeMo-co and the residual NMF solvent were separated from the ^{15}N-core-NafY/FeMo-co complex by applying at least five cycles (20-fold dilution per cycle) of concentration and dilution with anaerobic buffer C in an Amicon cell device equipped with a YM10 membrane (Millipore). NMF was estimated to be below 0.000003% at the end of the procedure.

Protein assays

SDS-PAGE was performed according to Laemmli [12]. Protein concentration in the samples (Table S5) was determined by UV-Vis spectroscopy at 280 nm or by the bicinchoninic acid method (BCA reagent, Pierce) using bovine serum albumin (BSA) as a standard [13]. Samples were pre-treated with iodoacetamide before performing the BCA assay to eliminate the interfering effect of DTH [14]. UV-visible spectroscopy was carried out in a Shimadzu (Kyoto) UV1601V spectrophotometer. The structure of core-NafY (1P90) in Fig. 1b was represented with PyMOL [15].

Computational modelling and molecular dynamics (MD) calculations

Possible binding sites were explored in protein-ligand docking calculations with AutoDock Vina [16] and Chimera 1.15 [17]. The crystal structure 1P90 of core-NafY was used for the receptor and the geometry of FeMo-co (Fe_7S_9CMo-homocitrate) cluster in the crystal structure of NifDK from *A. vinelandii* (PDB 3U7Q) [18] was used for the ligand. After locating putative sites in the vicinity of H^{121}, the best docking solutions were selected for optimizing the geometry of the corresponding NafY/FeMo-co complexes using the CHARMM 3.6 force field (FF) [19] with the improved version 3.6m [20] for the protein. FF parameters for FeMo-co were prepared following the procedure by R. Björnsson [21, 22] (https://sites.google.com/site/ragnarbjornsson/mm-and-qm-mm-setup). The best energies corresponded to a FeMo-co binding site between H^{121} and C^{196} of NafY. This was the initial geometry for all-atom MD exploratory 10-ns simulations.

Three MD trajectories were obtained for the core-NafY/FeMo-co complex (*i*) in that initial geometry (trajectory 1), (*ii*) restraining only the presence of H^{121} (trajectory 2), and (*iii*) restraining only the presence of C^{196} (trajectory 3). All MD calculations were performed with the multicore CUDA version of NAMD 2.13 [23] in the Tesla V100 GPU of the high-performance computing CBGP cluster. The systems composed of protein, FeMo-co and a periodic solvation box with 14 Å spacing and the TIP3P model of water [24] were prepared with VMD 1.9.3 [25]. Sodium and chloride ions were added to counter the total charges of the systems setting a 0.150 M salt concentration. The particle-mesh Ewald summation method was employed for long-range electrostatics and a 10 Å cutoff was set for short-range non-bonded vdW interactions. Initial geometries in each MD run were first minimized at 5,000 conjugate-gradients steps, water was then equilibrated at 1 atm and 298 K for 100 ps at Δt = 2 fs, and 10-ns trajectories were then obtained also at Δt = 2 fs (5 million steps) in the NPT ensemble at 1 atm and 298 K. T and P control was treated by means of the Langevin dynamics (T) and Nosé-Hoover Langevin piston method (P). NAMD output was saved every 5,000 steps to render trajectories composed of 1,000 frames that were processed and analyzed with VMD 1.9.3 [25].

The complete sequence of NafY consisting of 243 amino acids was modelled with Robetta[26] using the TrRefineRosetta[27] method. This procedure predicted five "best" (highest scores) models in which the core segment 90-221 has the same architecture as in the crystal structure 1P90. Four of these five models agree in predicting nearly identical geometries for both the N-terminal domain (residues 1-98) and the C-terminal 222-243 segment composed of an α-helix (residues 225-243) linked to the core domain through a short loop (residues 222-224).

Poisson-Boltzmann (PB) electrostatic potentials $V(\mathbf{r})$ were computed using the nonlinear PB equation with the APBS 1.5[28] plug-in implemented in PyMOL 2.4.0[15]. Sequential focusing multigrid calculations in 3D grids with step size 0.5 Å and 190 x 164 x 173 = 5,390,680 points at 0.150 M NaCl concentration and 310 K were performed in the final structure of MD trajectory 1 as well as in the best Robetta structure

(“model 3”) of the modelled complete NafY protein. Numerical output grids of $V(\mathbf{r})$ were mapped onto the molecular surface of proteins computed and rendered with PyMOL 2.4.0. $V(\mathbf{r})$ is given in units of kT/e, k being Boltzmann’s constant, T absolute temperature 310 K, and e unit electron charge.

Table S1. Attenuation of spins resonances in core-NafY upon FeMo-co binding. "Ratio" means intensity ratios between FeMo-co-bound and FeMo-co-less measurements. Gaps denote that the FeMo-co-bound data is ambiguous or that the resonance was unassigned. Ratios equal to zero represent true complete attenuation of the signal.

Residue	Sequence	Ratio	Residue	Sequence	Ratio	Residue	Sequence	Ratio
100	ARG		144	ARG		188	HIS	
101	VAL		145	SER		189	PRO	
102	PRO		146	THR	0.2559	190	LEU	
103	GLU		147	LEU	0.1439	191	LYS	
104	GLY	0.0576	148	ASP	0.1375	192	LYS	0.0558
105	SER	0.0461	149	VAL	0.0000	193	PRO	
106	ILE		150	ALA	0.0000	194	LYS	0.0726
107	ARG	0.0000	151	LEU	0.0000	195	GLY	0.0109
108	VAL	0.0928	152	ALA	0.1626	196	CYS	0.0000
109	ALA	0.0853	153	GLU	0.1926	197	ALA	0.1063
110	ILE	0.0000	154	ASP	0.0000	198	ALA	0.0869
111	ALA	0.0000	155	LYS	0.0000	199	GLN	0.1531
112	SER		156	ASN		200	GLU	
113	ASN		157	ALA		201	ALA	0.1628
114	ASN		158	TRP		202	ILE	0.2839
115	GLY		159	ARG	0.0000	203	ALA	
116	GLU		160	VAL	0.0676	204	GLU	0.5233
117	GLN		161	GLU		205	LEU	0.0441
118	LEU	0.0000	162	GLN	0.0000	206	GLN	0.1209
119	ASP		163	ILE	0.1389	207	THR	0.1576
120	GLY	0.0880	164	GLN	0.0923	208	VAL	0.0957
121	HIS	0.0000	165	ASP	0.1319	209	MET	
122	PHE	0.1080	166	CYS	0.1080	210	ALA	0.1157
123	GLY	0.1366	167	GLN	0.1141	211	GLY	0.1054
124	SER	0.0705	168	VAL		212	SER	
125	CYS	0.0000	169	LEU	0.0979	213	PRO	
126	LEU	0.0988	170	TYR	0.1200	214	PRO	
127	ARG	0.4355	171	VAL	0.0986	215	PRO	
128	PHE	0.0932	172	VAL		216	TRP	
129	LEU	0.2427	173	SER	0.0000	217	LEU	
130	VAL	0.0755	174	ILE		218	ALA	0.1413
131	TYR	0.0714	175	GLY	0.1348	219	LYS	0.2094
132	GLN	0.0749	176	GLY	0.0000	220	LEU	0.0800
133	VAL	0.0696	177	PRO		221	VAL	
134	SER	0.0978	178	ALA		222	GLY	0.1200
135	ALA	0.0935	179	ALA	0.0332	223	VAL	0.1435
136	LYS	0.0937	180	ALA		224	SER	0.1854
137	ASP	0.1162	181	LYS		225	ALA	0.2395
138	ALA	0.1476	182	VAL		226	GLU	0.1842
139	SER	0.1360	183	VAL	0.0553	227	GLU	
140	LEU	0.1505	184	ARG		228	ARG	
141	VAL		185	ALA	0.0768	229	VAL	
142	ASP		186	GLY		230	ARG	
143	ILE		187	ILE		231	PHE	

Table S2. NafY wild type (WT) and site-directed variants generated for this work.

NafY variant	Strain Name
WT	pN2LP52
H121L	pN2LP53
C196A	pN2LP54
H121L/C196A	pN2LP55

Table S3. Primer sequences used for overlapping PCR reactions.

NafY variant	Oligonucleotide sequence	Number
H121L	5´-CAACTGGATGGACTCTTCGGCTCCTGC-3´	105
	5´-GCAGGAGCCGAAGAGTCCATCCAGTTG-3´	106
C196A	5´-GAAGCCCAAGGGTGCCGCGGCGCAGGAGGC-3´	166
	5´-GCCTCCTGCGCCGCGGCACCCTTGGGCTTC-3´	167
H121L/C196A	5´-GAAGCCCAAGGGTGCCGCGGCGCAGGAGGC-3´	166
	5´-GCCTCCTGCGCCGCGGCACCCTTGGGCTTC-3´	167

Table S4. Primer combinations and PCR conditions for PCR 1 and PCR 2.

NafY variant	Template	PCR 1		PCR 2	
		Primers	Tm (°C)<br>Size (bp)	Primers	Tm (°C)<br>Size (bp)
H121L	*nafY* (WT)	2660<br>106	65.5<br>394	105<br>2661	68.1<br>408
C196A	*nafY* (WT)	2660<br>167	72<br>620	166<br>2661	72<br>182
H121L/C196A	*nafY* (H121L)	2660<br>167	72<br>620	166<br>2661	72<br>182

Table S5. Purified NafY variants used in this study.

Protein	Yield (mg/g cells)	Yield (mg/ml)
WT	0.94	6.72
H121L	1.35	9.65
C196A	0.32	2.34
H121L/C196A	0.13	0.95

Table S6. Mean root mean square deviations values (RMSD) and the corresponding standard deviations (σ) along MD simulations for the three MD trajectories of the core-NafY/FeMo-co complex. Protein values refer to backbone atoms and homocitrate (HCA) values refer to non-hydrogen atoms. All values (Å) computed upon aligning the protein structure with respect to the initial optimized geometry.

	**Protein**		**HCA**		**Fe_7S_9CMo moiety**	
	**RMSD**	**σ**	**RMSD**	**σ**	**RMSD**	**σ**
Trajectory 1	2.177	0.408	2.346	0.525	2.396	0.658
Trajectory 2	1.827	0.275	2.177	0.648	1.845	0.631
Trajectory 3	2.155	0.344	20.15	7.172	10.85	4.122

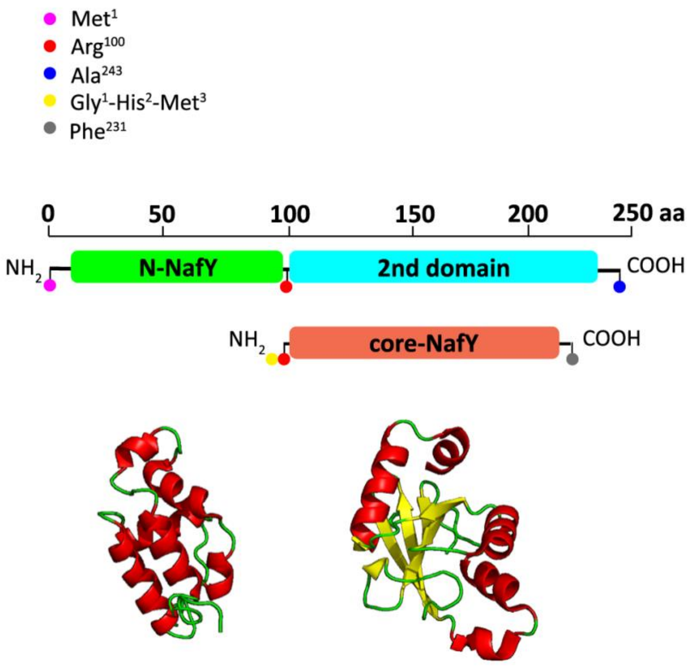

Fig. S1 Schematic representation of NafY first, second, and core domains. The core-NafY polypeptide used in this work, contains 132 amino acids (Arg^{100}-Phe^{231}) plus three N-terminal residues (Gly-His-Met) introduced during the cloning procedure.

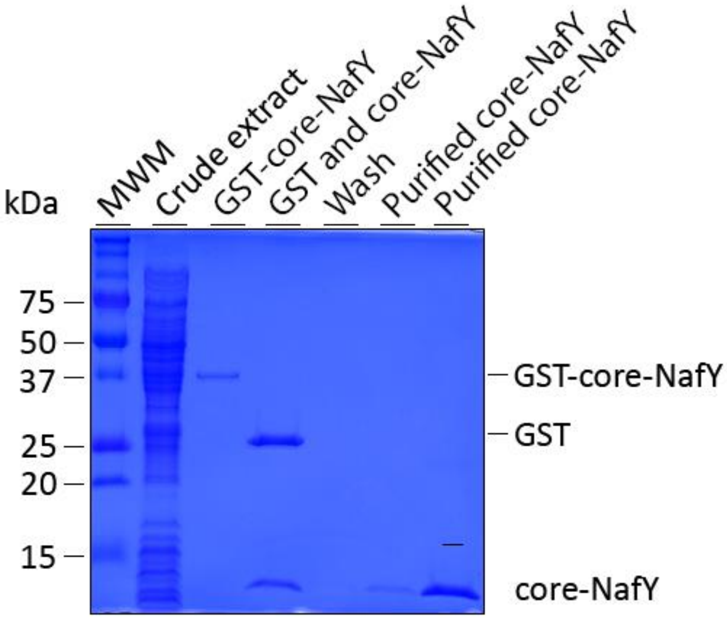

Fig. S2 SDS-PAGE analysis of ^{15}N-core-NafY purification steps. Cell-free extract lane contains 50 μg of total protein; purified protein lanes contain 1 and 7 μg of protein, respectively. *A. vinelandii* ^{15}N-core-NafY was expressed and purified from *E. coli* cells. Molecular mass markers are indicated to the left. The position of ^{15}N-core-NafY in the gel is indicated to the right.

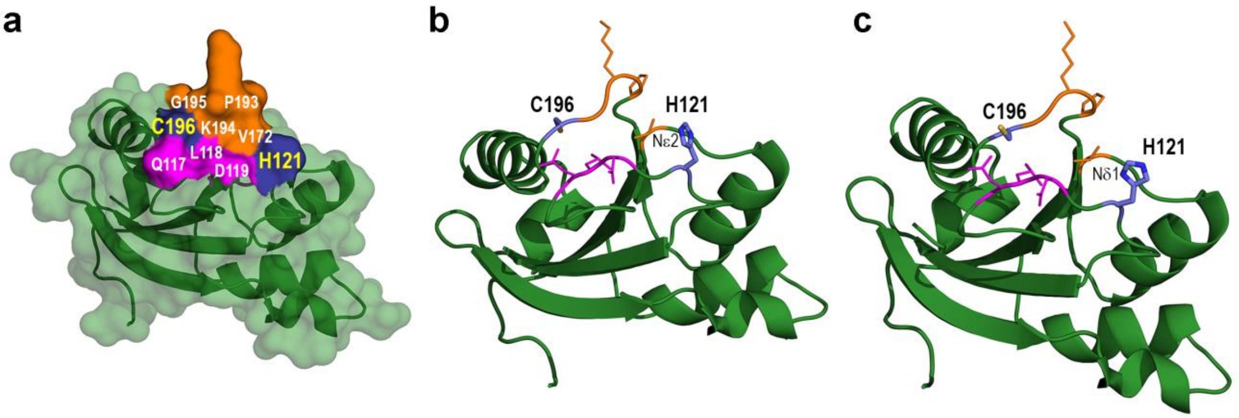

Fig. S3 Binding site in core-NafY located in molecular modelling. (a) Surface of the exposed cleft defined by the labelled residues found as putative binding site in exploratory docking calculations in the crystal structure of core-NafY (1P90). Magenta surface patch corresponds to Q117+L118 (β2 strand) + D119 (loop β2-β3: see Fig. S7) whereas orange surface patch corresponds to V172 (loop β5-α2) + P193+K194+G195 (loop β6-β7) and residues H^{121} and C^{196} are in blue. (b) Side chains of residues defining the surface cleft in the crystal structure. Rotamers of H^{121} and C^{196} are not properly oriented for binding FeMo-co. (c) Side chains of residues in (b) in the final structure after 10-ns MD simulation (trajectory 1) with rotamers of H^{121} and C^{196} properly oriented for binding.

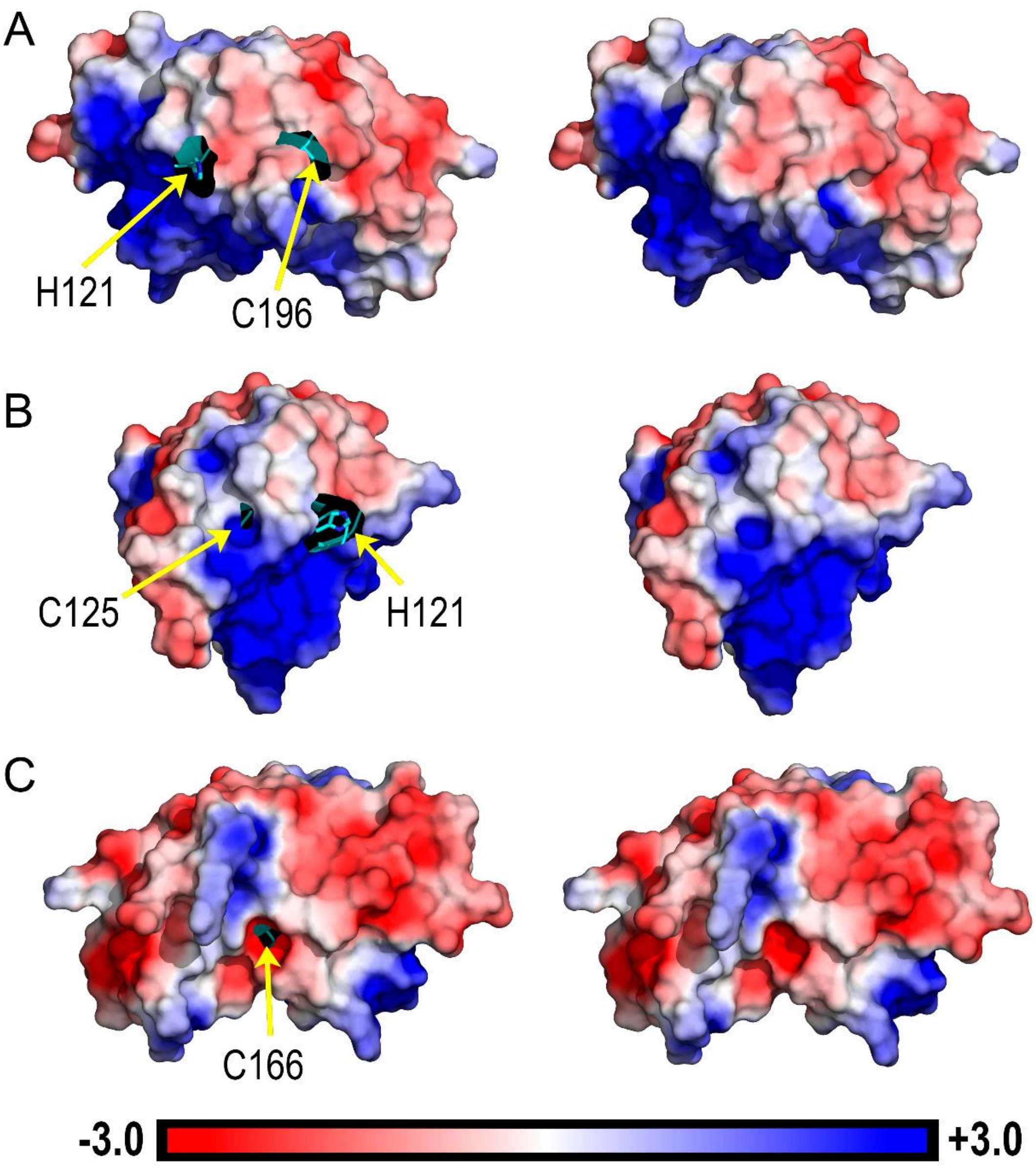

Fig. S4 Poisson-Boltzmann electrostatic potential *V*(**r**) mapped onto the molecular surface of core-NafY in the final structure after 10-ns MD simulation of its complex with FeMo-co. Left views hide the surface patches that correspond to the residues labelled. Right views show the complete surface at the same orientation as in left views. (A) The FeMo-co binding site is the concave surface patch with dominant negative *V*(**r**) between H^{121} and C^{196}. (B) Surface region between H^{121} and the nearby cysteine C^{125} shapes a convex patch with dominant neutral/positive *V*(**r**). (C) Tiny surface patch in the bottom of a small pocket with negative *V*(**r**) that corresponds to the largely buried C^{166} (the third cysteine of core-NafY) located at the opposite surface side of the FeMo-co binding site. Surfaces colored according to the scale bar with values of *V*(**r**) in *kT*/*e* units.

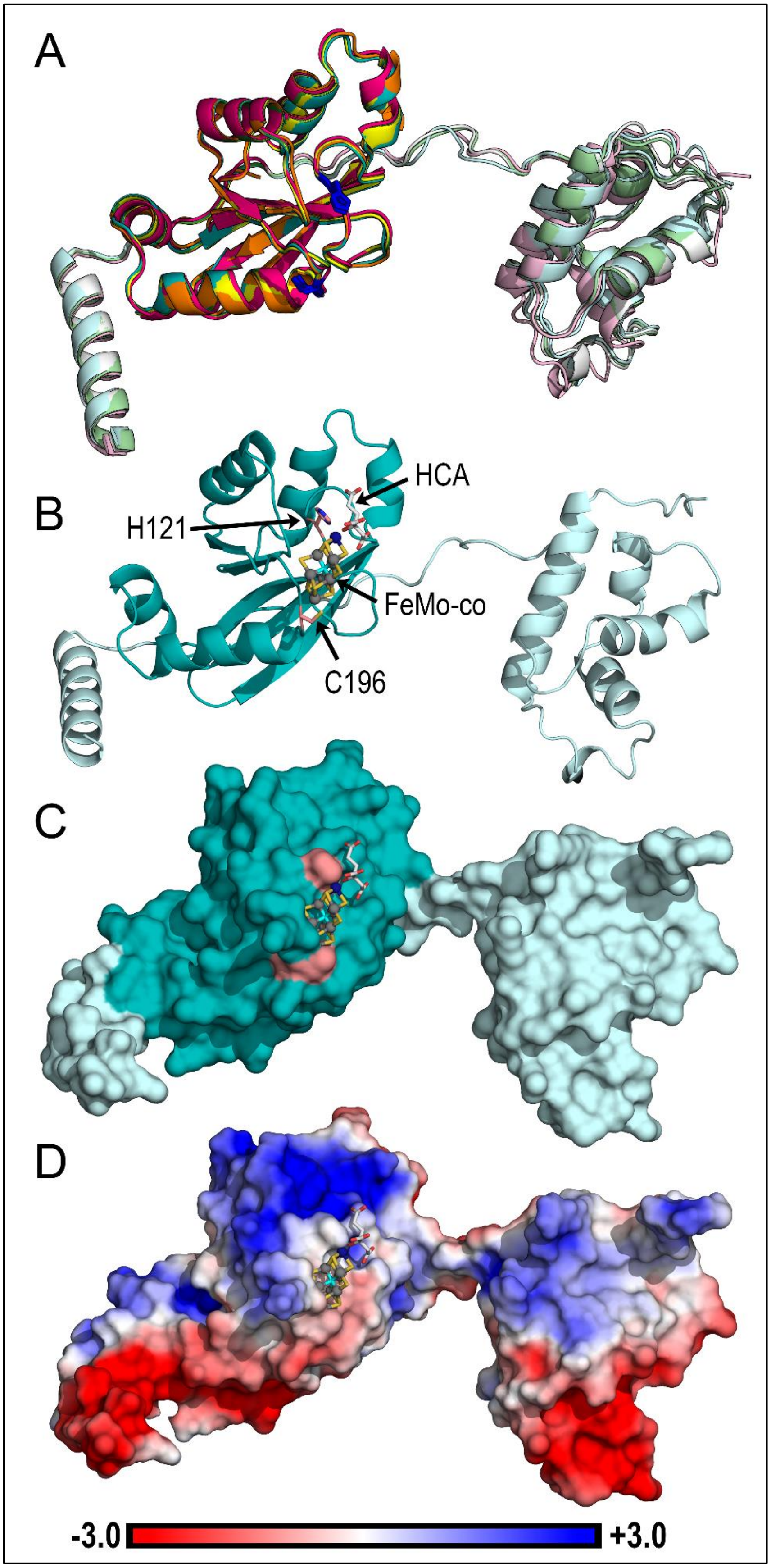

Fig. S5 Model structure of the complete 243-amino acids NafY protein. (A) Superposition of four Robetta models (green, yellow, cyan, and pink cartoons) that agree in predicting the same architecture for all the domains and the crystal structure 1P90 of core-NafY (orange cartoon). Light hues are used for N-terminal 1-98 segments (right domain) and for C-terminal 222-243 segments (left helical domain), while deep hues are used for the core 99-221 segment (middle domain). Side chains of H^{121} and C^{196} residues are shown as blue sticks. (B) Best, highest score Robetta model with FeMo-co ligands shown at the geometry corresponding to the superposition with crystal structure of core-NafY shown in (A). HCA means homocitrate. (C) Molecular surface of model shown in (B) with FeMo-co depicted as sticks. Salmon color indicates surface patches corresponding to C^{196} (left) and H^{121} (right). (D) Poisson-Boltzmann electrostatic potential *V*(r) mapped onto the surface shown in (C) colored according to the scale bar with values of *V*(r) in *kT*/*e* units.

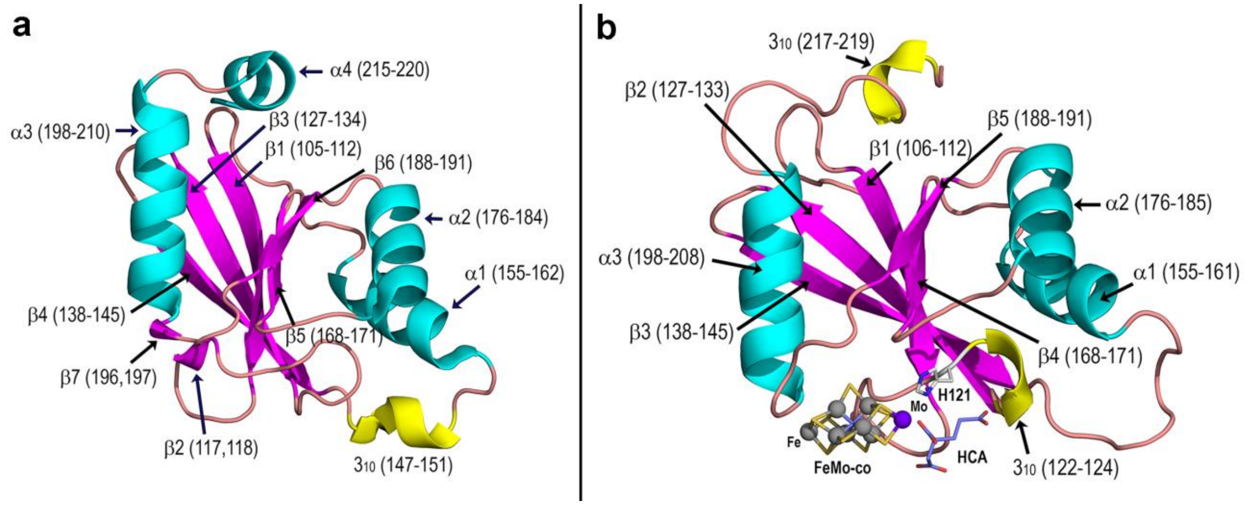

Fig. S6 Secondary structure of core-NafY identified with DSSP [29]. (a) Crystal structure of core-NafY (1P90). (b) Final structure after 10-ns MD simulation of the complex with FeMo-co in trajectory 1. Side chain of His^{121} is also shown as sticks with carbon atoms in white and nitrogen atoms in blue.

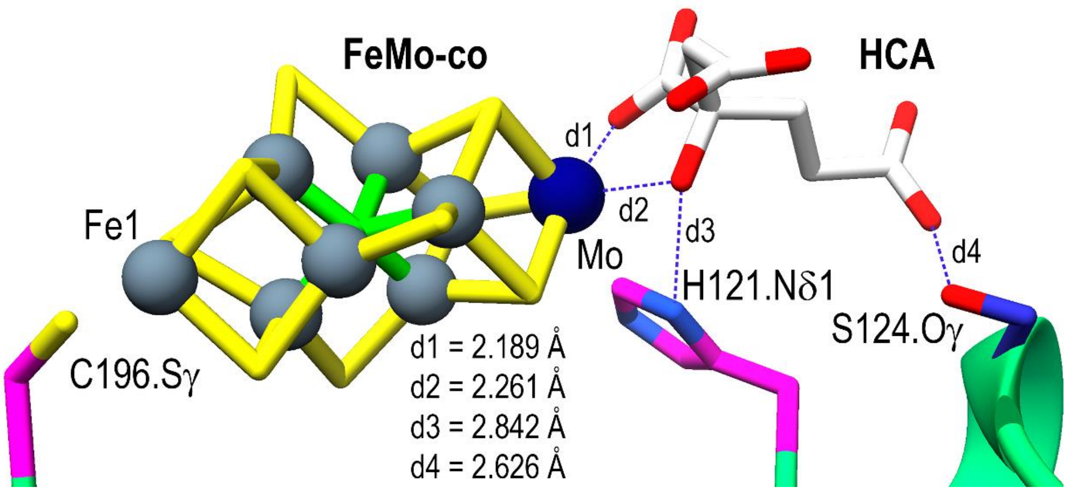

Fig. S7 Neighborhood of 4 Å around any atom of homocitrate (HCA) in the final structure after 10-ns MD simulation of the complex of core-NafY with FeMo-co.

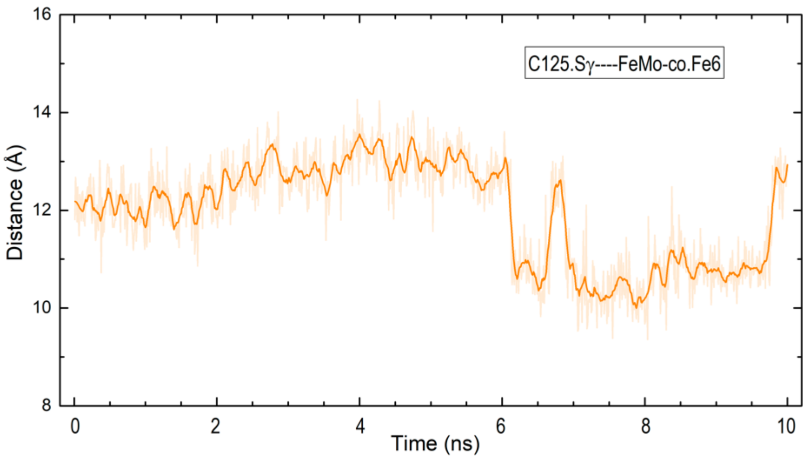

Fig. S8 Change of C^{125}.Sγ – FeMo-co.Fe6 distance. All-atom MD 10-ns simulations of core-NafY/FeMo-co complex were carried out to calculate the distances.

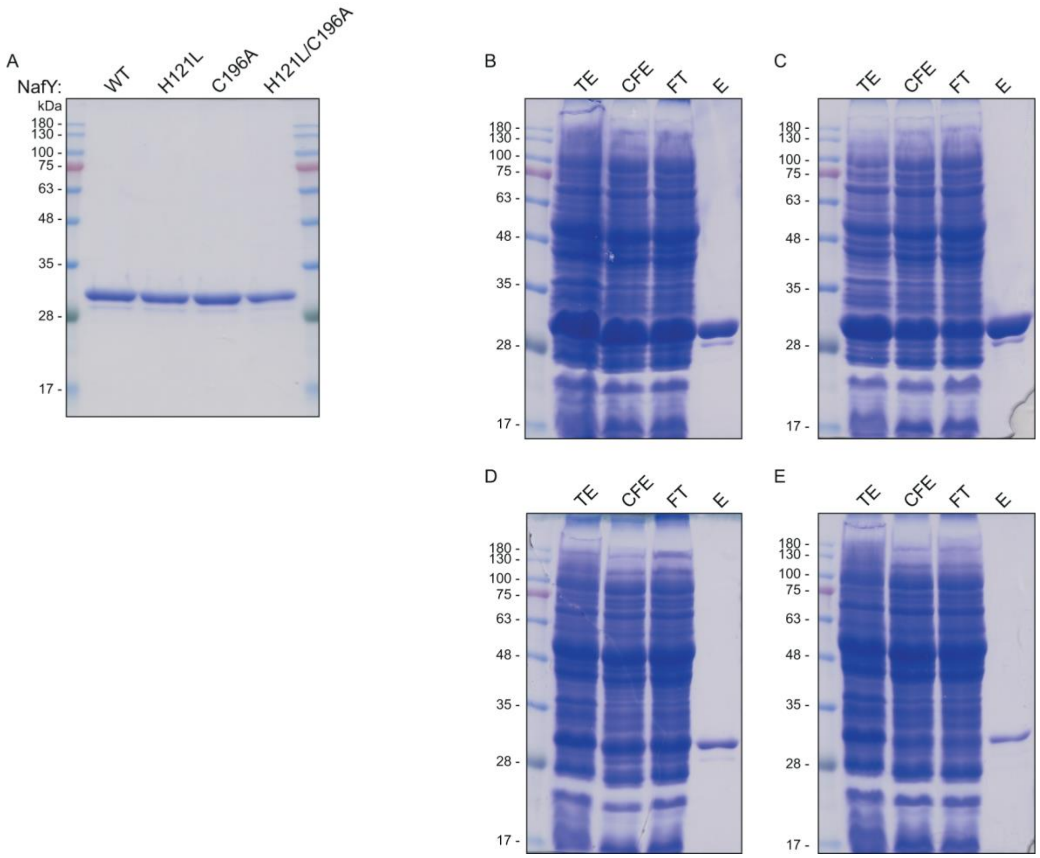

Fig. S9 SDS-PAGE analysis of NafY variants purification processes. (A) Purified preparations of NafY WT and site-directed variants. Purification steps of NafY wild type (B), H121L (C), C196A (D), and H121L/C196A, (E). TE, total extract. CFE, cell-free extract. FT, flow-through. E, elution. Molecular weight markers are indicated to the left.

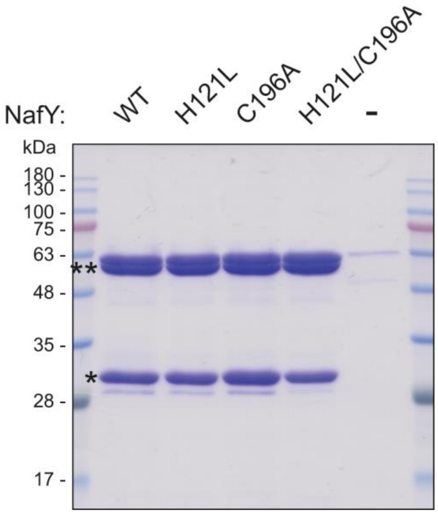

Fig. S10 Analysis of NafY variant interaction with apo-NifDK. SDS-PAGE analysis of *A. vinelandii* UW146 proteins eluted from NafY pull-down experiments showing apo-NifDK (**) and NafY (*) bands. The negative control (-) shows elution from resin not previously loaded with NafY. Molecular masses are indicated to the left.

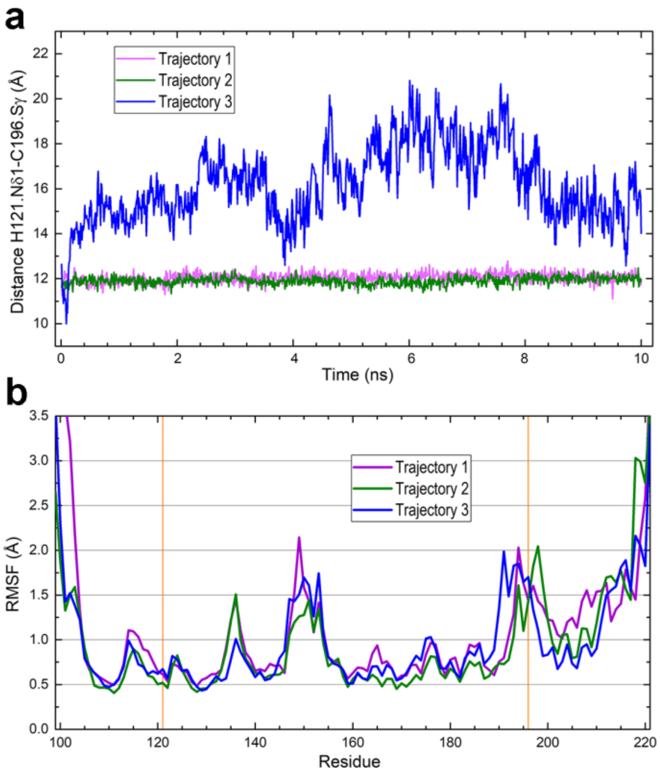

Fig. S11 Comparison of output for the three MD trajectories of the core-NafY/FeMo-co complex starting at the following initial structures. Trajectory 1: optimized geometry of the complex, trajectory 2: ibid. restraining only the presence of H^{121}, and trajectory 3: ibid. restraining only the presence of C^{196}. (a) Change along the MD simulations of the distance between Nδ1 atom of H^{121} and Sγ atom of C^{196}. (b) Root mean square fluctuation of Cα atoms. Orange vertical lines indicate H^{121} and C^{196} residues.

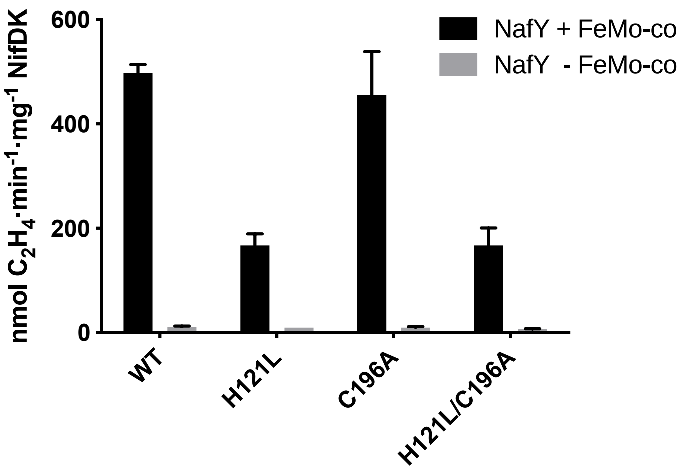

Fig. S12 Activation of apo-NifDK by NafY/FeMo-co complexes. Samples contained 0.6 µM apo-NifDK and 1.2 µM NafY (alone or in complex with FeMo-co). Values represent average activity ± SD (n=2).

References

1. J. S. a. D. W. Russell, *Molecular cloning. A laboratory manual*, Cold Spring Harbor Laboratory Press, Cold Spring Harbor, N.Y., Third edn., 2001.
2. J. Marley, M. Lu and C. Bracken, *J. Biomol. NMR*, 2001, **20**, 71-75.
3. S. N. Ho, H. D. Hunt, R. M. Horton, J. K. Pullen and L. R. Pease, *Gene*, 1989, **77**, 51-59.
4. D. H. Dyer, L. M. Rubio, J. B. Thoden, H. M. Holden, P. W. Ludden and I. Rayment, *J. Biol. Chem.*, 2003, **278**, 32150-32156.
5. E. V. Koskela and A. D. Frey, *Mol. Biotechnol.*, 2015, **57**, 233-240.
6. J. A. Hernandez, A. H. Phillips, W. K. Erbil, D. Zhao, M. Demuez, C. Zeymer, J. G. Pelton, D. E. Wemmer and L. M. Rubio, *J. Biol. Chem.*, 2011, **286**, 6321-6328.
7. R. M. Allen, M. J. Homer, R. Chatterjee, P. W. Ludden, G. P. Roberts and V. K. Shah, *J. Biol. Chem.*, 1993, **268**, 23670-23674.
8. L. M. Rubio, S. W. Singer and P. W. Ludden, *J. Biol. Chem.*, 2004, **279**, 19739-19746.
9. F. Delaglio, S. Grzesiek, G. W. Vuister, G. Zhu, J. Pfeifer and A. Bax, *J. Biomol. NMR*, 1995, **6**, 277-293.
10. R. Keller, ETH Zurich, 2005.
11. V. K. Shah and W. J. Brill, *Proc. Natl. Acad. Sci. U. S. A.*, 1977, **74**, 3249-3253.
12. U. K. Laemmli, *Nature*, 1970, **227**, 680-685.
13. P. K. Smith, R. I. Krohn, G. T. Hermanson, A. K. Mallia, F. H. Gartner, M. D. Provenzano, E. K. Fujimoto, N. M. Goeke, B. J. Olson and D. C. Klenk, *Anal. Biochem.*, 1985, **150**, 76-85.
14. H. D. Hill and J. G. Straka, *Anal. Biochem.*, 1988, **170**, 203-208.
15. W. Delano, *Journal*.
16. O. Trott and A. J. Olson, *J. Comput. Chem.*, 2009, **31**, 455-461.
17. E. F. Pettersen, T. D. Goddard, C. C. Huang, G. S. Couch, D. M. Greenblatt, E. C. Meng and T. E. Ferrin, *J. Comput. Chem.*, 2004, **25**, 1605-1612.
18. T. Spatzal, M. Aksoyoglu, L. Zhang, S. L. Andrade, E. Schleicher, S. Weber, D. C. Rees and O. Einsle, *Science*, 2011, **334**, 940-940.
19. A. D. MacKerell, D. Bashford, M. Bellott, R. L. Dunbrack, J. D. Evanseck, M. J. Field, S. Fischer, J. Gao, H. Guo, S. Ha, D. Joseph-McCarthy, L. Kuchnir, K. Kuczera, F. T. Lau, C. Mattos, S. Michnick, T. Ngo, D. T. Nguyen, B. Prodhom, W. E. Reiher, B. Roux, M. Schlenkrich, J. C. Smith, R. Stote, J. Straub, M. Watanabe, J. Wiorkiewicz-Kuczera, D. Yin and M. Karplus, *J. Phys. Chem. B*, 1998, **102**, 3586-3616.
20. R. B. Best, X. Zhu, J. Shim, P. E. Lopes, J. Mittal, M. Feig and A. D. Mackerell, Jr., *J. Chem. Theory Comput.*, 2012, **8**, 3257-3273.
21. B. Benediktsson and R. Bjornsson, *Inorg. Chem.*, 2017, **56**, 13417-13429.
22. C. Van Stappen, A. T. Thorhallsson, L. Decamps, R. Bjornsson and S. DeBeer, *Chem. Sci.*, 2019, **10**, 9807-9821.
23. J. C. Phillips, D. J. Hardy, J. D. C. Maia, J. E. Stone, J. V. Ribeiro, R. C. Bernardi, R. Buch, G. Fiorin, J. Henin, W. Jiang, R. McGreevy, M. C. R. Melo, B. K. Radak, R. D. Skeel, A. Singharoy, Y. Wang, B. Roux, A. Aksimentiev, Z. Luthey-Schulten, L. V. Kale, K. Schulten, C. Chipot and E. Tajkhorshid, *J. Chem. Phys.*, 2020, **153**, 044130.
24. W. L. Jorgensen, J. Chandrasekhar, J. D. Madura, R. W. Impey and M. L. Klein, *J. Chem. Phys.*, 1983, **79**, 926-935.
25. W. Humphrey, A. Dalke and K. Schulten, *J. Mol. Graph.*, 1996, **14**, 33-38, 27-38.
26. D. E. Kim, D. Chivian and D. Baker, *Nucl. Acids. Res.*, 2004, **32**, W526-W531.
27. J. Yang, I. Anishchenko, H. Park, Z. Peng, S. Ovchinnikov and D. Baker, *Proc. Natl. Acad. Sci. U. S. A.*, 2020, **117**, 1496-1503.

28. N. A. Baker, D. Sept, S. Joseph, M. J. Holst and J. A. McCammon, *Proc. Natl. Acad. Sci. U. S. A.*, 2001, **98**, 10037-10041.
29. W. Kabsch and C. Sander, *Biopolymers*, 1983, **22**, 2577-2637.
